# Computational assessment of the retinal vascular tortuosity integrating domain-related information

**DOI:** 10.1038/s41598-019-56507-7

**Published:** 2019-12-27

**Authors:** L. Ramos, J. Novo, J. Rouco, S. Romeo, M. D. Álvarez, M. Ortega

**Affiliations:** 1 University of A Coruña, Department of Computer Science and Information Technology, A Coruña, Spain; 2 CITIC-Research Center of Information and Communication Technologies, University of A Coruña, A Coruña, Spain; 3 Grupo VARPA, Instituto de Investigación Biomédica de A Coruña (INIBIC), Complexo Hospitalario Universitario de A Coruña (CHUAC), Sergas, Universidade da Coruña (UDC), As Xubias, 15006 A Coruña, Spain; 4 Servizo de Oftalmoloxía, Complexo Hospitalario Universitario de Ferrol, Ferrol, A Coruña, Spain

**Keywords:** Computational science, Diagnostic markers, Computer science, Medical imaging, Software

## Abstract

The retinal vascular tortuosity presents a valuable potential as a clinical biomarker of many relevant vascular and systemic diseases. Commonly, the existent approaches face the tortuosity quantification by means of fully mathematical representations of the vessel segments. However, the specialists, based on their diagnostic experience, commonly analyze additional domain-related information that is not represented in these mathematical metrics of reference. In this work, we propose a novel computational tortuosity metric that outperforms the mathematical metrics of reference also incorporating anatomical properties of the fundus image such as the distinction between arteries and veins, the distance to the optic disc, the distance to the fovea, and the vessel caliber. The evaluation of its prognostic performance shows that the integration of the anatomical factors provides an accurate tortuosity assessment that is more adjusted to the specialists’ perception.

## Introduction

Retinal vascular tortuosity can represent a potential indicator of a significative number of vascular and systemic diseases such as diabetic retinopathy, cerebrovascular disease, stroke, and ischemic heart disease^1–4^. However, the manual characterization of the vascular tortuosity is affected by several factors that limit its use for diagnostic and treatment purposes, added to the tedious and time-consuming process that it typically involves. The main limitation identified in systematic reviews conducted by Kalitzeos *et al*.^5^ and Abdalla *et al*.^6^ is the lack of a precise and standard guide for characterizing the retinal vascular tortuosity. Therefore, in the clinical practice, the manual tortuosity assessment is mostly based on the experience of the expert clinicians in identifying abnormal structural characteristics in the vessels, variations in their normal course, or dissimilarities to normal healthy vessels in terms of width, length, or number of turns. This subjective appreciation of the vessel tortuosity entails a high inter and intra expert variability. Despite this, clinical findings stated that different tortuosity effects are dependent on different diseases as obstructive sleep apnea, diabetes, diabetic retinopathy, or cerebral vascular diseases^7–10^, as reference. For that reason, the tortuosity characterization should be analyzed considering the specific underlying disease. Furthermore, there are also relevant limitations related to the availability of unified public datasets or the lack of a standard regarding the image acquisition, the algorithm for the extraction of the retinal vascular tree, or the relevant regions of the fundus image for the tortuosity assessment^5,6^.

In the literature, there are several approaches for the computational tortuosity assessment, mostly based on mathematical representations using the vessel centerlines. Among them, Hart *et al*.^11^ presented the most intuitive tortuosity measurements, incorporating concepts like the ratio between the length and the chord of the vessel or the total curvature. The authors also formalized several properties to integrate partial measurements in a global tortuosity metric for the whole retinal image. In subsequent works, Bhuiyan *et al*.^12^ introduced some changes considering numerical derivatives for the calculus of the total curvature, whereas Trucco *et al*.^13^ generalized the measurement of the vessel skeleton curvature proposed by Hart *et al*.^11^. Another approach proposed by Grisan *et al*.^14^ incorporated the times that a vessel curves by splitting each vessel in *n* segments of constant-sign curvature, combining and integrating posteriorly the evaluation of such segments. A similar idea was proposed by Onkaew *et al*.^15^, which also incorporated an improved chain-code algorithm to compute the target curvature.

Previous works conducted by Sánchez *et al*.^16,17^ performed a preliminary validation of the metrics of reference over a standard set of retinal images that was manually classified as tortuous/non-tortuous. Subsequent works were conducted in^18,19^ with the aim of advancing in the standardization of the vascular tortuosity as a clinical biomarker with diagnostic potential. In these works, a complete and exhaustive multi-expert validation was performed in order to establish a consistent clinical criteria to validate the effectiveness of the objective computational metrics of reference. This evaluation included the manual annotations provided by a group of five specialists that are actively involved in the daily clinical practice of an ophthalmological service. The different experience levels of the specialists, from the head of the service to resident physicians, are aimed to encompass the full spectrum of expert knowledge. In order to use a representative cohort of homogeneous and consistent data, the dataset was limited to diabetic patients given the strong association between vascular tortuosity and diabetes and, in particular, diabetic retinopathy. A rating procedure composed of different rating rounds and a posterior consensual session was designed in order to clarify the discrepancies, gain consensus and establish unified criteria for the tortuosity characterization. Finally, the prognostic performance of the objective metrics of reference was evaluated in relation to the specialists performance.

The results extracted from these studies^18,19^ show that, although the metrics of reference provide acceptable results, they do not supply a full representation of the perception of the specialists. Among the metrics of reference, the proposal of Grisan *et al*.^14^ provided the closest results to the specialists performance. However, as indicated, these metrics are exclusively based in mathematical properties of the vessel segments, stating the tortuosity degree on the basis of one or two factors such as the curvature, the amplitude, or the number of turns and twists, depending on each case. Despite that, there are additional domain-related parameters that do not strictly correspond to the vessel course but the specialists, based on their experience, also consider for the assessment of the tortuosity. The computational metrics of reference do not still incorporate, at the moment, these parameters, thereby causing some distance between the automated effectiveness and the specialist perception. Therefore, anatomical factors such as the vessel caliber^20^, the location or zone of the tortuous vessels^5^, or the distinction between arteries and veins^6,21^ have been identified as relevant for the tortuosity assessment. Thus, a computational metric that provides a tortuosity characterization more useful and reliable to support the clinical decision-making process should incorporate additional anatomical properties of the fundus image that better represent the clinical analysis.

In this work, a new computational metric to measure the retinal vascular tortuosity is proposed, combining the accurate mathematical tortuosity representation with representative clinically derived knowledge. In particular, to this end, the metric proposed by Grisan *et al*.^14^ that provided the best prognostic performance in previous analyses^18,19^ was set as the baseline metric. This metric provides a mathematical representation of the tortuosity of the vessels segments. Additionally, as result of several meetings with the specialists, a set of anatomical factors were identified as relevant to influence the vascular tortuosity assessment. These anatomical properties include the distinction between arteries and veins, the distance to the optic disc, the distance to the fovea, and the vessel caliber. To the best of our knowledge, the validity and importance as well as the incorporation of each of these parameters for the tortuosity assessment have not been previously analyzed.

The computational metric consists of a set of progressive steps to compute the global tortuosity measurement of the whole analyzed retina. To this end, firstly the arterio venous tree is extracted from the input fundus image, being its constituent vessels divided into individual segments. Then, a local tortuosity value is computed for each vessel segment by means of its mathematical properties as well as the incorporation os the different considered anatomical factors. Finally, the global tortuosity for the whole retina is obtained from the weighted composition of the tortuosity values computed for each vessel composing the retinal vascular tree. With the aim of evaluating the impact of the incorporation of the anatomical factors, the effectiveness of the proposed metric was compared to the effectiveness of the baseline mathematical metric of reference, demonstrating the improvement of the novel proposed tortuosity metric in relation with the performance of the specialists.

This work is not aimed to provide a global metric that replaces the subjective expert evaluation. Instead, it is intended to establish the basis for advancing in the standardization of the retinal vascular tortuosity as a clinical biomarker with diagnostic potential, allowing, thereby, objective computational measurements that better represent the analysis performed by the specialists in the clinical practice. These advances should help improving the very low agreement that there is among specialists regarding tortuosity assessment.

This paper is structured as follows: Section presents the designed dataset and describes the proposed metric for tortuosity assessment including the anatomical factors identified by the specialists. Next, Section exposes all the conducted experiments whereas Section discusses the obtained results. Finally, Section presents the conclusions and possible future work.

## Materials and Methods

### Dataset

The used dataset contains 200 retinal images of patients that present any diabetes grade varying from non visible signs of abnormal vascular course to severe tortuosity. The data used in this study have been previously anonymized. The project DTS15/00153 that includes this study and its corresponding informed consent was approved by the “Comité de Ética da Investigación de A Coruña-Ferrol” committee belonging to the “Rede Galega de Comités de Ética da Investigación” attached to the regional government “Secretaría Xeral Técnica da Consellería de Sanidade da Xunta de Galicia” (Ref. 2015/501) and conducted in accordance with the tenets of the Helsinki Declaration.

The lack of a precise and standard guide for assessing the retinal vascular tortuosity leads to a subjective appreciation of the analyzed data. In order to have a representative ground-truth covering the entire spectrum of the expert knowledge, the dataset was manually annotated by a group of five specialists that are actively involved in the daily clinical practice of an ophthalmological service, from the head of the service to resident physicians. In previous studies^18,19^, a complete and exhaustive inter-intra multi-expert analysis showed that the lack of standard criteria entails a low agreement between the specialists. Therefore, in these studies, in order to clarify discrepancies, gain consensus and establish unified criteria for the tortuosity characterization, a procedure comprising two rating rounds and a joint session involving the whole set of specialists was performed over a subset of 60 fundus images. This analysis was initially performed according to a qualitative four-grade from non-tortuous to severe tortuousity, being complemented with non-tortuous/tortuous and asymptomatic/symptomatic binary classifications. The evaluation of the suitability of these scales led to decide the use of the asymptomatic/symptomatic binary classification given its relevance for clinical practice. In this work, in order to prevent misleading, the term asymptomatic/symptomatic has been renamed to relevant/non-relevant, considering non-relevant those cases where there is no sign of tortuosity or there is mild asymptomatic tortuosity whereas relevant corresponds to cases with moderate or severe tortuosity that can be associated to significant risks.

Once these consensual criteria were established, the remaining images were manually rated by each of the 5 specialists. Prior and during the rating procedure, the specialists were explicitly instructed to adhere exclusively to the assessment of the retinal vascular tortuosity, omitting other clinical findings such as the presence of microaneurysms, hemorrhages, drusen, etc. that could originate biased rates. Each of the specialists rated individually the dataset in a blind process without any available information on the patient medical condition.

Table 1 summarizes the manual rates indicated by the set of specialists $E=\{{E}_{1},{E}_{2},..\,,{E}_{5}\}$ on the basis of the binary classification relevant/non-relevant for the whole dataset. Despite the labels provided for each expert, a set of consensual labels *R*_*c*_ was obtained from the most voted label for each included retinal image.

**Table 1:** Distribution of the manual annotations provided by 5 different specialists in the whole dataset using the binary classification relevant/non-relevant.

	E1	E2	E3	E4	E5	Rc
0:non-relevant	159	132	128	109	136	141
1:relevant	41	68	72	91	64	59

The last column *R*_*c*_ corresponds to the distribution of the most voted label for each retinal image.

### Automatic measurement of the retinal vascular tortuosity

**Figure 1:**
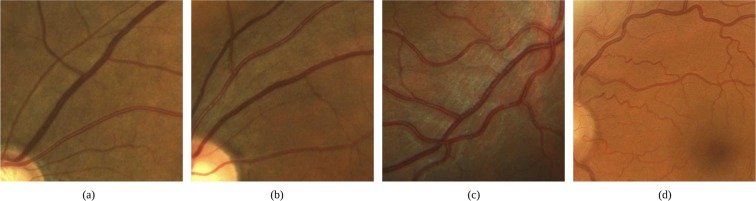
Retinal images with (**a**,**b**) non-tortuous and (**c**,**d**) tortuous blood vessels.

Retinal vascular tortuosity is characterized by an abnormal curvature of the vessels, evincing a non-smooth appearance, presenting turns and twists throughout their course. Some representative examples of fundus images with non-tortuous and tortuous blood vessels are shown in Fig. 1. In the literature, there are several computational metrics that provide a global quantification of the retinal vascular tortuosity by means of mathematical properties of the vessel segments such as the curvature, the ratio between the arch and the chord or the number of turns. However, the previously conducted analysis of the metrics of reference^18,19^ outcomes that the specialists, based on their experience, analyze a larger set of anatomical properties of the fundus image that are not only represented in these pure mathematical metrics. Therefore, a computational tortuosity characterization that represents the expert perception should integrate further anatomical factors apart from the mathematical representation of the vessel tortuosity.

**Figure 2:**
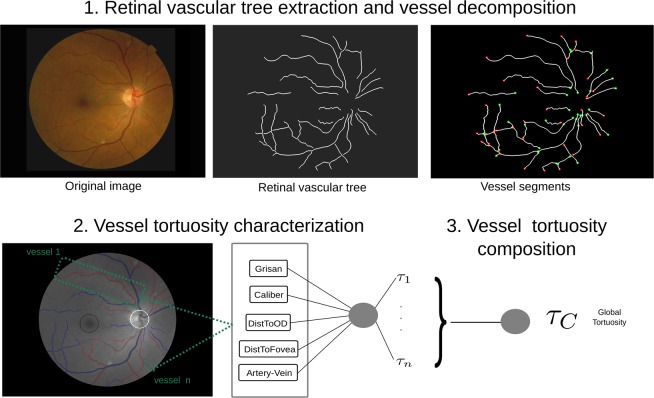
Steps for the computational tortuosity measurement. The first block is in charge of the extraction of the arterio-venous tree and the decomposition into its constituent vessels. In the second block, a local tortuous value is computed for each vessel. In the third block, the local tortuosity values are combined to compute the global tortuosity quantification.

As a result of several meetings with the specialists, a set of anatomical factors were identified as relevant to influence the vascular tortuosity assessment. Therefore, on one hand, considering that different diseases produce different tortuosity effects in the arterio-venous tree, the *distinction between arteries and veins* is considered relevant for the tortuosity assessment. On the other hand, noting that the main vessels are thicker than the rest of the vessels, the *caliber* is considered an indicator of the vessel relevance in the global estimation. Furthermore, tortuous vessels around the optic disc or the fovea may have more clinical significance, so the *distance to the optic disc* as well as the *distance to the fovea* are also considered as relevant factors for the tortuosity assessment. This work proposes to incorporate these domain-related parameters to the computational tortuosity measurement with the aim of obtaining an evaluation closer to the expert perception. The proposed metric computes a global estimation of the retinal vascular tortuosity by means of an analysis involving the whole retinal vascular tree. Therefore, the proposed computational metric consist of a tortuosity quantification based on specific mathematical criteria combined with the integration of the mentioned anatomical factors to weight each vessel tortuosity value throughout the tortuosity vessel composition process. This metric is composed by the steps illustrated in Fig. 2. Firstly, there is an initial stage in charge of the extraction of the arterio-venous tree and the division into its constituent vessels. Then, each vessel is examined individually in terms of both its mathematical properties and the considered anatomical factors. The information extracted from this analysis is combined in order to compute a tortuosity value for each retinal vessel composing the vascular tree. Finally, the tortuosity values extracted for each individual vessel are integrated in order to obtain the global tortuosity quantification for the whole retina.

#### Retinal vascular tree extraction and vessel decomposition

The initial stage of the methodology takes as input the original retinal image and extracts the set of the vessel segments composing the arterio-venous tree. Therefore, the extraction of the retinal vascular tree is performed by means of a crease based algorithm^22,23^. In particular, it consists in the detection of the blood vessels from the ridges and valleys in the retinal image, this is, the regions that form a tubular pattern sharply defined on the neighborhood. To this end, the Multi Local Set of Extrinsic Curvature enhanced by the Structure Tensor (MLSEC-ST) operator is applied to detect the vessels from the ridge lines. Then, a thinning process is performed to extract the centerline of a maximum of 1 *px* width for each vessel^24^. After this, an edge tracking algorithm is applied to decompose the vessel tree into its constituent vessels. Finally, the vessel point coordinates are locally smoothed in order to minimize the discrete effect of the pixel representation.

#### Vessel tortuosity characterization

Once the vessel segments composing the retinal vascular tree are extracted, each one is individually examined to compute the local segment tortuosity. This local tortuosity characterization is performed in terms of a mathematical representation of the involved vessel segment and also of the integration of the identified anatomical factors in order to approach the knowledge of the specialists. The metric proposed by Grisan *et al*.^14^ is used as the baseline mathematical representation given that among the computational metrics of reference, this is the one that provides the prognostic performance closest to the specialist perception^18,19^. The anatomical factors used for the local tortuosity computation are the *distinction between arteries and veins*, the *caliber*, the *distance to the optic disc* and the *distance to the fovea*. The local vessel tortuosity is obtained from a weighted combination of these parameters.

**Figure 3:**
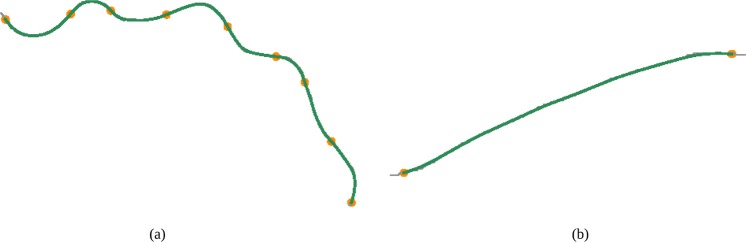
Grisan Vessel decomposition into the constituent subsegments of constant-sign curvature.

Mathematical vessel characterization - Grisan *et al*.^14^. In order to compute the mathematical vessel representation proposed by Grisan *et al*.^14^, the vessel is subdivided in *n* segments of constant-sign curvature, as shown in Fig. 3. Then, the evaluation of such segments and their amount are combined as follows:

1 $${\tau }_{g}=\frac{n-1}{{L}_{c}}\,\mathop{\sum }\limits_{i=1}^{n}\,[\frac{{L}_{csi}}{{L}_{xsi}}-1]$$

where *L*_*c*_ corresponds to the arc length of the vessel whereas *L*_*csi*_ and *L*_*xsi*_ represent the arc length and the chord length of each subsegment. This metric integrates the information about how many times a vessel changes convexity so that a higher number of subsegments implies higher tortuosity.

Distance to the optic disc. The optic disc is seen as a significative bright circular region where the retinal blood vessel tree emerges. The main motivation indicates that vascularity close to the optic disc is more relevant that others more distant. In particular, the detection of the optic disc is based on the location of a circular bright shape on the fundus image^25^. To this end, first, a luminosity and contrast normalization is applied to reduce the illumination effects. Then, a DoG filter is applied for the location of the region of interest. After that, the Canny edge detector is used for the extraction of the edges into the region of interest. These edges include pixels corresponding to both the optic disc and the vessels. In order to discard the edge points related to the vessels, the points located near a crease line belonging to the arterio-venous tree are discarded. Finally, the fuzzy Hough transform is applied using only the optic disc edge points. Additionally, a segmentation step is performed to fit the optic disc boundaries and, thereby, to improve the detection. Once the optic disc is detected, the distance to the optic disc is computed using the Euclidean distance between the center of the circle that defines the optic disc and the center point of each vessel segment.

Distance to the fovea. The fovea is a small depression located at the center of the macula, which can be seen as the darkest region of the fundus image. It is responsible for the sharp central vision, for what the retinal vessels around this regions have a significative clinical relevance. Given the morphological properties of the eye, the size and the position of the previously detected optic disc are used for the macula detection. Therefore, in order to detect the macula, firstly, a candidate region is defined as a circular area centered at approximately 2 disc diameters away from the optic disc center and the double size of the optic disc^26^. Then, a correlation filter which consists of a Laplacian of Gaussians is applied to the region in order to locate the macula, and so, the fovea, which is located at the position where the filter presents the maximum response^26^. In the same way as the distance to the optic disc, the distance to the fovea is computed using the euclidean distance between the center of the fovea and the center point of each vessel.

Distinction between arteries and veins. Given that different diseases are related to different vascular effects, the distinction between arteries and veins represent meaningful information for the tortuosity assessment. The artery-vein classification is computed from the retinal vascular tree and the center of the optic disc that was extracted in the previous steps. For each vessel composing the vascular tree, a set of points of interest is selected to classify them into arteries or veins. Then, the values computed at each point of interest of a vessel are integrated to obtain an unique label of artery or vein. Given the inherent radial structure of the vascular tree, these points of interest are selected from the intersection of concentric equidistant circumferences centered at the optic disc and the vessel centerlines. Given these intersection points, deformable models (snakes) are used for the vessel segmentation^27^. The deformation starts at each of these points and evolves under the influence of several energies based on image features until the snake points reach the vessel boundaries. Then, a topological adjustment is applied to avoid deviations in the snake contour. This segmentation process results in a shape similar to a parallelogram surrounding the vessel segment. For each parallelogram, 1-pixel lines perpendicular to the vessels are selected as the profiles from which the feature vectors will be extracted for the classification. In order to enhance the image before extracting the feature vectors, a multi-scale retinex technique^28^ is applied to the original image. The best features that were found for the classification are the median of the green channel of the multi-scale retinex output at each profile. The method for the vessel labeling consists in dividing the retinal image into overlapping areas in order to minimize the influence of lightness variations. Then, the feature vectors that were extracted at each of these areas are classified as arteries or veins, and finally, the local classifications are combined in order to obtain the final class of each vessel segment^28,29^. Additionally, a procedure to track the vessel segments along the vessel in the different circumferences is performed to avoid possible misclassifications^30^.

Vessel caliber. The distinction between the main and the secondary vessels, which is given by its thickness, is also considered by the specialists for the tortuosity assessment and, thereby, the vessel caliber is also relevant as an anatomical factor. Therefore, the parallelograms computed in the previous step are used for measuring the vessel caliber at each point of interest and, then, the vessel caliber for each segment is computed as the mean of the caliber values of all the points of interest that were selected for this segment^27^.

Integration of mathematical and anatomical factors. Optimization using an evolutionary multi-objective algorithm. Motivated by the set of anatomical factors that were identified as relevant to influence the vascular tortuosity assessment, this work is aimed to combine these anatomical factors with the mathematical vessel representation in order to get an unique tortuosity measurement for each retinal vessel that better represents the perception of the specialists. Given that the relative influence of each of these factors is unknown, this combination is performed by means of a weighted linear combination of all the factors in which the weights are free parameters that are optimized to predict the perception of the specialists. Thus, this model was chosen as the simplest generic one including all the factors.

To this end, a weight is applied to each anatomical factor and the local tortuosity for each vessel *ci* is computed as follows:

2 $${\tau }_{c}={\tau }_{{g}_{c}}{f}_{c}$$

where ${\tau }_{{g}_{ci}}$ is the mathematical representation based on the Grisan metric according to Eq. 1 and *f*_*ci*_ is the weighted factor computed from the different anatomical factors and the vessel length according the Eq. 3

3 $$\begin{array}{ccc}{f}_{c} & = & [({\omega }_{AV}\ast (1-{c}_{AV})+(1-{\omega }_{AV})\ast {c}_{AV})+({\omega }_{caliber}\ast {c}_{caliber})\\  &  & +\,({\omega }_{dOD}\ast {c}_{dOD})+({\omega }_{dFov}\ast {c}_{dFov})]\ast {L}_{c}\end{array}$$

where ${\omega }_{AV}$, ${\omega }_{caliber}$, ${\omega }_{dOD}$ and ${\omega }_{dFov}$ are the weights assigned to the distinction between arteries and veins (${c}_{AV}$), the caliber (${c}_{caliber}$), the distance to the optic disc (${c}_{dOD}$) and the distance to the fovea (${c}_{dFov}$), respectively, whereas *L*_*c*_ is the length of the vessel.

In order to obtain the optimal configuration for the combination of these parameters according their corresponding weights, a multi-objective optimization process is performed. The Nondominated Sorting GA approach (NSGA)^31^ was selected for this purpose given its accurate performance. The objective of the NSGA algorithm is to improve the adaptive fit of a population of candidate solutions to the Pareto front constrained by a set of objective functions by means of an evolutionary process which implies evolutionary operators including selection, genetic crossover, and genetic mutation. Additionally, the optimization methodology follows statistical cross-validation procedures, with separate training and test sets at the patient level, which ensures that the provided results are independent of the model selection and fitting biases. Thus, this methodology ensures that the study, along with the conclusion derived from the fitted models, is statistically sound, independently of the retrospective nature of the data gathering.

#### Vessel tortuosity composition

Finally, the tortuosity values that were computed for each vessel composing the vascular tree are integrated in a global tortuosity measurement by a weighted additivity. This way, the computed global tortuosity is associated as a single score for the whole retina. Therefore, using the compositionality property^11^ in (1), each vessel contributes directly proportional to its arc length as follows:

4 $${\tau }_{C}=\frac{{\sum }_{i=1}^{n}\,{\tau }_{ci}{f}_{ci}}{{\sum }_{i=1}^{n}\,{f}_{ci}}$$

where *f*_*ci*_ is the computed weight for the vessel *ci* and ${\tau }_{ci}$, the tortuosity value for that vessel.

## Experiments and Results

In order to assess the agreement between the specialists in the manual rating, an overall comparison was carried out including all the manual rates that were provided by the five specialists for the entire dataset. This analysis shows that there is a full consensus among all the specialists in the 56.5% of the images composing the dataset and a percentage of 79.5% of images in which at least four specialists agreed in their labels. The remaining 20.5% of the images present higher controversy with an agreement of three specialists that marked one label versus the other two specialists that marked the opposite. Additionally, Cohen-Kappa indexes^32^ were computed between each pair of specialists as well as between each specialist and the consensus *R*_*c*_. The results are showed in Table 2.

**Table 2:** Cohen-Kappa indexes for inter-rater agreement as well as between each specialist and the consensus *R*_*c*_ for relevant/non-relevant classification.

Cohen-Kappa	E2	E3	E4	E5	*R*_*c*_
E1	0.47	0.56	0.41	0.33	0.55
E2		0.54	0.58	0.53	0.69
E3			0.62	0.58	0.79
E4				0.58	0.65
E5					0.71

With the aim of evaluating the prognostic performance of the computational tortuosity assessment, a ROC (Receiver Operating Characteristic) analysis^33^ was performed. For this purpose, the consensual rates *R*_*C*_ were set as the target predictions, corresponding to the most voted label according to the relevant/non-relevant binary classification. Thus, ROC curves can be built from the reciprocal relation between sensitivity and specificity calculated for all the possible threshold values in the computational metrics. In order to evaluate the impact of integrating the anatomical factors, this analysis was carried out both for the baseline metric that uses pure mathematical representations and for the proposed metric, that also incorporates the identified anatomical factors. Additionally, the same ROC analysis was also used to evaluate the performance of each specialist with respect to the consensual rates *R*_*c*_. This way, a point in the ROC space is obtained for each specialist by means of the sensitivity and specificity that were obtained by comparing the corresponding labels. This allows to compare the prognostic performance of the computational metrics with respect to the specialists performance.

In order to provide a reliable validation of the methodology ensuring its generalization capability, Monte Carlo Cross-Validation^34^ was used in the performed experiments. For this purpose, 10 independent random splits of the dataset into training and test data were created with the same percentage of non-relevant and relevant cases in each one of them. For each split, the optimization of the weighted factors was performed over the training data, whereas the predictive performance was assessed over the test data. In this way, the optimization of the weighted factors and the evaluation of the optimized models were performed over independent sets. Then, the obtained ROC curves were interpolated over the splits and the Area Under the Curve (AUC)^33^ was used to obtain a reliable representation of the generalization capability of the proposed method. Thus, this ensures that the study results are statistically sound and, indeed, validating the optimization of the weighted factors, independently of the retrospective nature of the data gathering.

For this purpose, on one hand, ROC curves were built for the baseline metric ${\tau }_{G}$ in the training and test sets. On the other hand, the equivalent analysis was performed for the proposed metric ${\tau }_{C}$. To this end, the training process consisted of a multi-objective optimization by means of the NSGA algorithm^31^ to set the configuration for combining the anatomical factors with the mathematical tortuosity representation. This multi-objective optimization was focused on maximizing the sensitivity and the specificity with respect to the consensual rates *R*_*C*_. The parameters involved in the evolutionary process were, on one hand, the weights ${\omega }_{AV}$, ${\omega }_{caliber}$, ${\omega }_{dOD}$ and ${\omega }_{dFov}$, corresponding to each anatomical factor, and, on the other hand, a threshold *t*_*h*_ over the tortuosity values obtained by applying these weights in Eq. 4. Therefore, each candidate solution in the evolutionary process allows to classify each fundus image of the dataset according to the relevant/non-relevant binary classification, providing, thereby, a point in the ROC space from the reciprocal relation between sensitivity and specificity^33^. Then, a ROC curve was built from the convex hull of the set of points provided by the different candidate solutions. After the training process, the candidate solutions that are part of the Pareto front were applied to the test set, and a ROC curve was built from the convex hull of the results provided for the test set in the ROC space. Theses processes were repeated over each of the partitions of the dataset, and the obtained ROC curves were interpolated to obtain a global result for each of the metrics ${\tau }_{G}$ and ${\tau }_{C}$ in the training and the test stages.

**Figure 4:**
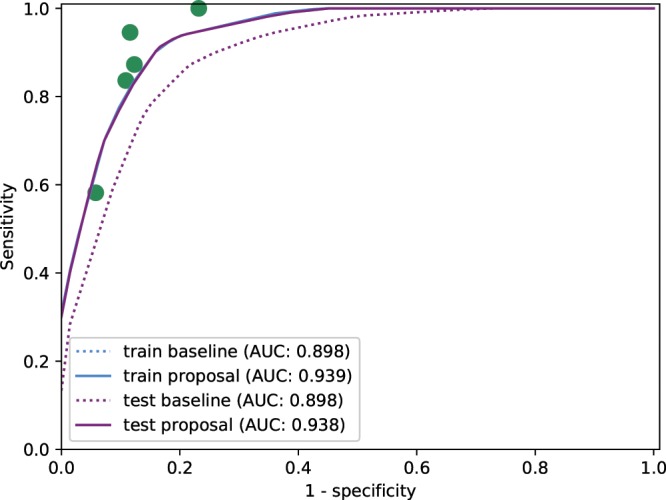
ROC curves for the baseline metric ${\tau }_{G}$ and the proposed metric ${\tau }_{C}$ that includes the anatomical factors in the training and test processes.

Figure 4 shows the global ROC curves obtained for the mathematical baseline metric ${\tau }_{G}$ and the proposed metric ${\tau }_{C}$ throughout the training and test stages. The dot marks represent the performance of the specialists with respect to the consensual rates *R*_*c*_. The graphs show that the integration of the anatomical factors outperforms the prognostic performance of the baseline metric based on pure mathematical representations of the vessel segments.

**Figure 5:**
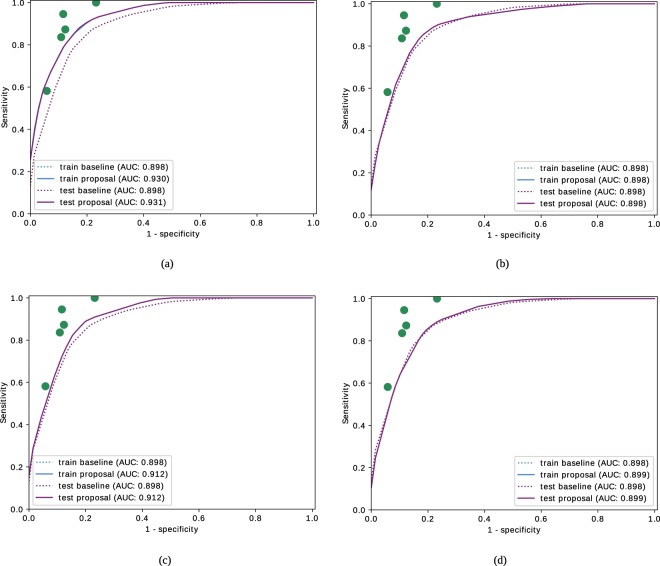
ROC curves for the baseline metric and proposed metric using independently each individual factor in the training and test process. The considered anatomical factors are (**a**) the distinction between arteries and veins, (**b**) the distance to the optic disc, (**c**) the distance to the fovea and (**d**) the vessel caliber.

In order to analyze the clinical relevance of each anatomical factor independently from the rest, the process described above is repeated by incorporating each factor separately in the evolutionary optimization process. The graphs in Fig. 5 show the results obtained for each one of the anatomical factors. These results point that the distinction between arteries and veins is the most relevant anatomical factor to assess the tortuosity, followed by the distance to the fovea. The vessel caliber and the distance to the optic disc does not report an individual significative contribution, however, they may be relevant in combination with the rest of the factors.

**Figure 6:**
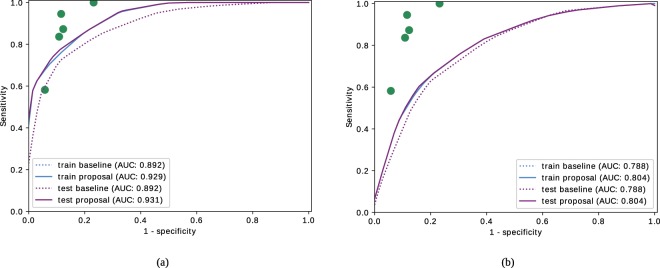
ROC curves for the baseline metric and proposed metric considering separately (**a**) arteries and (**b**) veins in the training and test process.

Given the relevance of the distinction between arteries and veins, an additional analysis considering separate tortuosity indexes for arteries and veins was also performed. To this end, ROC curves obtained for the mathematical baseline metric ${\tau }_{G}$ and the proposed metric ${\tau }_{C}$ are performed separately for the vessel segments labelled as arteries (Fig. 6(a)) and for the vessel segments labelled as veins Fig. 6(b) throughout the training and test stages. This analysis reveals that the tortuosity index of the arteries provides a representation significantly closer to the subjective perception of the specialists than the tortuosity index of the veins. Although the tortuosity index of the veins provides results farther from the perception of the specialists, it may report some contribution combined with the tortuosity index of arteries.

**Figure 7:**
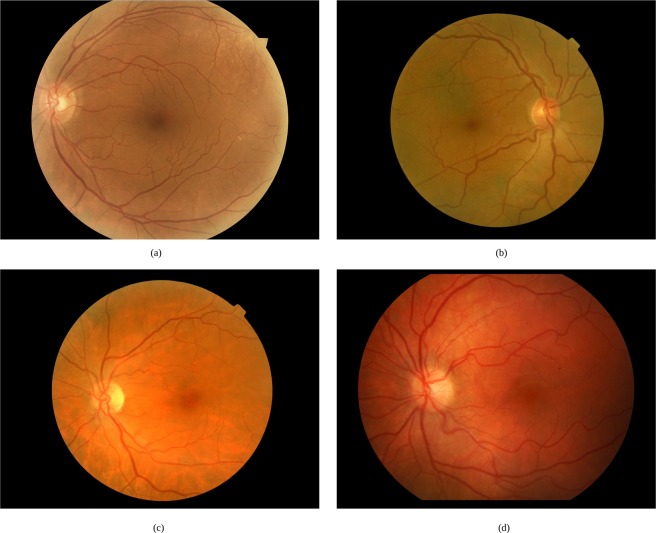
Examples of representative cases where the incorporation of the anatomical factors allows to produce a correct tortuosity characterization.

Additionally, in order to perform a qualitative evaluation of the impact of the incorporation of the anatomical factors, specific configurations that provided the best compromise between specificity and sensitivity throughout the training process were selected for the baseline metric ${\tau }_{G}$ that is only based on the mathematical representation of the vessel tortuosity and the vessel length, and for the proposed metric ${\tau }_{C}$, that also incorporates the distinction between the arteries and veins, the vessel caliber, the distance to the optic disc and the distance to the fovea. Some representative examples of the application of these configurations over retinal images of the used dataset are presented in Fig. 7.

## Discussion

The overall comparison among the specialists shows that there is a significant percentage of retinal images in which at least four specialists agree in their labels. Regarding the Cohen-Kappa indexes, the standard guidelines for interpreting the values^35^ assumes a substantial agreement over 0.61. Although some specialists present fair or moderate agreement among them, the agreement in relation to the consensual rates is substantial for the specialists labeled as *E*_2_ to *E*_5_ and moderate for the specialist *E*_1_.

The evaluation of the prognostic performance of the computational metrics shows that the baseline metric provides acceptable results, but remaining at a distance of the specialists performance. Regarding the proposed metric, the incorporation of the anatomical factors provides a substantial improvement, allowing a performance equivalent to some of the specialists that rated the dataset. The analysis by each specific anatomical factor shows that the distinction between arteries and veins and the distance to the fovea played the main role to approximate the tortuosity characterization to the perception of the specialist. Although the caliber and the distance to the optic disc does not present an individual significative contribution, they may be relevant combined with the rest of the factors. This way, the combination, for example, between the caliber and the distinction between arteries and veins allows to give more relevance to the main veins.

The analysis considering separately the tortuosity indexes for arteries and veins showed that the perception of the specialists is mostly related with the tortuosity index of the arteries. However, although the tortuosity index of the veins provides results farther from the perception of the specialists, the combination of both indexes implies, in general, an improvement in the prognostic performance of the tortuosity characterization.

A qualitative analysis of the impact of of integrating the anatomical factors was performed over specific configurations of the baseline metric and the proposed metric. In particular, Fig. 7(a) shows an example of a retinal image classified as relevant by the specialists. In this case, most of the tortuous vessels are secondary vessels that appear close to the fovea. However, there exists a significative number of main vessels that do not present signs of abnormal curvature. The result obtained by means of the baseline metric ${\tau }_{G}$ classified this image as non-relevant. The tortuosity values computed for the tortuous vessels are diluted in the vessel composition process when they are combined with other vessels with lower tortuosity values. However, the proposed metric ${\tau }_{C}$ gives more weight to those vessels close to the fovea, so that the result after the vessel composition indicates that the image is relevant, in line with the labels of the specialists.

Figure 7(b) shows another representative example of a relevant image in which the most tortuous vessels are the main veins as well as some secondary vessels around the fovea. In this case, the classification provided by the baseline metric ${\tau }_{G}$ is in the limit between relevant and non-relevant whereas ${\tau }_{C}$ provides a clear relevant label given the higher weight of the veins with high caliber and secondary vessels close to the fovea.

Figure 7(c) represents a case of a relevant retina with a few tortuous vessels that are mainly located close to the optic disc or close to the fovea. The baseline mathematical tortuosity computation provides a false negative rate whereas the incorporation of these anatomical factors allows to correctly classify the image as relevant. Similarly, Fig. 7(d) represents a relevant case where there exists a number of normal vessels but the tortuous vessels present a significatively high caliber, being also located close to the optic disc or the fovea, so the anatomical factors incorporate to the computational metric the capacity to achieve a correct classification.

In general, the incorporation of the anatomical factors to the tortuosity computation supposes a performance improvement, especially in terms of avoiding many false negatives and also without carrying any penalization in other cases. Regarding the false positives, some cases occurred both in the baseline mathematical metric and in the proposed new metric. These cases are mainly derived by an incorrect vessel segmentation that provides a false continuity among the vessel and their ramifications that causes a false tortuosity effect. In this sense, a refinement and improvement of this previous step would allow a better performance since a proper vessel segmentation is critical for the tortuosity characterization.

## Conclusions

The retinal vascular tortuosity is related to several ocular and systemic diseases, for what a reliable tortuosity characterization presents a valuable potential for diagnostic ant treatment purposes. The lack of a precise and standard definition of the vascular tortuosity implies that the manual tortuosity characterization is conditioned by the subjective appreciation of the specialists, causing, therefore, a high inter and intra expert variability.

Several works in the literature propose different approaches for the computational tortuosity characterization. These metrics are mostly based in pure mathematical representations of the vessel segments to define the degree of tortuosity depending on factors such as the amplitude, the curvature or the number of involved turns. However, in the clinical practice, the specialists, on the basis of their experience, commonly analyze a larger set of properties that are not incorporated in the computational metrics. Therefore, the metrics of reference, although they provide acceptable results, do not cover a complete representation of the expert perception.

This work proposes the integration of several domain-related properties in order to perform a computational tortuosity characterization that better represents the expert criteria. For this purpose, a set of anatomical factors including the distinction between arteries and veins, the distance to the optic disc, the distance to the fovea, and the vessel caliber were identified as relevant properties for the tortuosity characterization throughout several meetings with a group of 5 specialists. One of the metrics of reference based on mathematical properties of the vessel segments was selected as the baseline mathematical metric. Despite this mathematical analysis, the set of additional anatomical characteristics was incorporated to the metric in order to weight the tortuosity value of each vessel according to them. Therefore, the tortuosity computation consists of an initial step in charge of the retinal vascular tree extraction and the decomposition into its constituent vessels. Then, each vessel segment is individually analyzed in terms of its mathematical properties as well as each of the identified anatomical factors. A multi-objective optimization process focused on maximizing the sensitivity and the specificity was used to set the optimal configuration for the combination of all these properties in an unique tortuosity value for each vessel. Finally, a composition process is performed to compute the global tortuosity measurement for the whole retina, using the partial tortuosity values that were obtained for each vessel composing the retinal vascular tree.

In order to evaluate the impact of the incorporation of this anatomical factors, the prognostic performance of the proposed metric was compared to the performance provided by the baseline metric, only based in the mathematical representation of the vessel segments. To this end, each of the metrics was evaluated over a dataset composed by 200 fundus images that were manually rated by a group of five specialists according to the relevant/non-relevant binary classification. The results confirm that the integration of the anatomical properties allows a better tortuosity characterization, closer to the expert perception. The main improvement lies on the decrease of the false negative rates since the anatomical factors give more weight to relevant tortuous vessels whereas in the baseline metric these values are diluted through the composition process. The independent analysis of each factor reveals that the distinction between the arteries and the veins played the main role in the improvement of the prognostic performance. However, although the caliber and the distance to the optic disc do not provide any improvement independently, the combination with the rest of the factors showed a slight increase in the prognostic performance.

Regarding the identified cases of false positives, both for the baseline mathematical metric and for the new proposed metric, the main limitations are related to the robustness of the vessel tree analysis. It has been observed that the ability to obtain a representative arterio-venous tree, as well as the subsequent division into vessel segments, can be compromised by varying image qualities and conditions. While this is not a limitation for the target study, which is the analysis of the relevant factors regarding tortuosity assessment, it has been identified as one of the most important issues preventing the proposed methods in reaching a higher agreement with the specialists. In this regard, future work in this research contemplate a refinement of the initial stages for arterio-venous segmentation and tree decomposition in order to obtain a better representation of the vascular structure, especially regarding the representative landmarks as crossings and bifurcations. In order to do this, the use of deep learning techniques to improve these steps is considered.

In parallel with this, the multidisciplinary research team of this work is carrying out the collection of additional data derived from the medical history that are not explicitly included in the retina, such as age, gender, cholesterol, blood pressure, etc. in order to perform complementary analyses for cardiovascular risk stratification.
